# FLAG/FLAG-Ida Regimen in Secondary and Relapsed/Refractory Acute Myeloid Leukemia—Even in the Era of New Treatment Modalities Still a Significant Player

**DOI:** 10.3390/jcm13071842

**Published:** 2024-03-22

**Authors:** Saša Anžej Doma, Matjaž Sever, Gorazd Jakoš, Helena Podgornik

**Affiliations:** 1 Hematology Department, University Medical Centre Ljubljana, Zaloška Cesta 2, 1000 Ljubljana, Sloveniahelena.podgornik@kclj.si (H.P.); 2 Faculty of Medicine, University of Ljubljana, Vrazov Trg 2, 1000 Ljubljana, Slovenia; 3 Faculty of Pharmacy, University of Ljubljana, Aškerčeva 7, 1000 Ljubljana, Slovenia

**Keywords:** relapsed/refractory AML, secondary AML, FLAG-Ida, FLAG, *FLT3* mutation

## Abstract

(1) **Background**: Relapsed/refractory (r/r) and secondary acute myeloid leukemia are highlighted by chemoresistance and poor outcomes. The aim of the study was to assess the efficacy and toxicity of fludarabine, cytarabine, and granulocyte-colony stimulation factor (FLAG) with or without idarubicin (-Ida) and to discuss novel therapies in this setting. (2) **Methods**: Clinical and cytogenetic data on 130 consecutive patients with r/r and secondary AML treated at our center were retrospectively analyzed. (3) **Results**: There were 48, 56, and 26 patients with relapsed, refractory, and secondary AML, respectively. The median age was 60 years. The overall response was achieved in 70% of patients. The median overall survival (OS) time for the whole group was 9.4 months. In total, 47% of patients proceeded to allogeneic hematopoietic stem cell transplantation (aHSCT) and these patients had significantly prolonged OS compared to the others (63 months vs. 4.2 months; *p* < 0.001). Among the variables, including age, *FLT3* mutation status, European LeukemiaNet (ELN) 2022 classification risk, FLAG vs. FLAG-Ida, and aHSCT, a multivariate analysis revealed that only aHSCT significantly influenced overall survival. (4) **Conclusions**: FLAG(-Ida) chemotherapy remains an effective salvage chemotherapy for patients with r/r and secondary AML with a plan of proceeding to aHSCT.

## 1. Introduction

Relapsed and refractory (r/r) acute myeloid leukemia (AML) represent one of the most challenging conditions in hematology, with a 5-year survival rate of only 10% [1]. The advent of targeted drugs (gilteritinib, ivosidenib, etc.) and small molecules (venetoclax) in recent years has led to a decrease in the use of high-dose salvage chemotherapy [2]. While this shift is particularly evident for older and unfit patients [3], the question remains whether younger, fit patients still benefit from intensive chemotherapy followed by allogeneic hematopoietic stem cell transplantation (aHSCT). The latter remains the only option with curative potential [4].

Secondary AML encompasses AML that arises from a previous myeloid hematologic disease or prior chemotherapy or radiotherapy administered for another condition. It is a subgroup of AML characterized by treatment resistance and poor outcomes [5]. The recent approval of CPX-351, a liposomal formulation of cytarabine and daunorubicin, for patients aged 60–75, demonstrated a survival advantage (9.56 vs. 5.95 months) over standard-of-care cytarabine plus daunorubicin (DA7+3) chemotherapy [6]. For younger patients, various therapeutic options, including high-intensity regimens combining nucleoside analogs and high doses of cytarabine, are employed for secondary AML [7]. Similar to r/r AML, secondary AML is also a high-risk disease that requires aHSCT for achieving durable remission [8].

The combination of fludarabine, cytarabine, and granulocyte-stimulating factor (FLAG) with or without idarubicin (-Ida) has been used for almost 25 years in r/r AML, with reported remission rates of 50–65% in cohorts of 45–221 patients [9,10,11]. However, due to their toxicity, FLAG and FLAG-Ida are being replaced with targeted therapies where applicable or combined with novel drugs [12]. Nevertheless, FLAG-Ida has been investigated also as a first-line treatment demonstrating a superior remission rate and reduced risk of relapse, but higher toxicity and no survival benefit compared to DA7+3 [13]. The studies of patients treated with FLAG-Ida as frontline therapy demonstrated response rates exceeding 80% but generally included younger patients [13,14,15].

The aim of our study was to evaluate the efficacy, safety, and outcomes of FLAG and FLAG-Ida regimens in the setting of r/r and secondary AML in patients treated at our center.

## 2. Materials and Methods

All adult patients with r/r AML and secondary AML who were treated with FLAG and FLAG-Ida between 1 January 2012 and 31 December 2022 at our center were retrospectively analyzed. Patients with acute promyelocytic leukemia were excluded.

Patient and disease characteristics at the beginning of treatment were collected. Risk assessment and disease response were assessed according to the latest European LeukemiaNet (ELN) criteria [16]. Due to retrospective analysis and a long observation period, we decided to use a consistent approach, taking into consideration all available molecular and cytogenetic data. Additionally, we collected data on the duration of hospitalization (defined as the time from the first day of chemotherapy until discharge from the hospital), early mortality, febrile episodes, and aHSCT.

The FLAG-Ida protocol consisted of fludarabine 30 mg/m^2^/day and cytarabine 2 g/m^2^/day for 5 consecutive days (4 days in additional cycle), during which idarubicin 8 mg/m^2^/day was administered on 3 consecutive days; in FLAG protocol, idarubicin was omitted [13]. Usually, only patients with good performance status (ECOG 0–2) were selected for treatment with this regimen. The decision on FLAG vs. FLAG-Ida was based on the patient’s comorbidities (cumulative anthracycline dose and cardiac failure) and the physician’s preference. G-CSF was given 1 day prior to chemotherapy for 7 consecutive days and again after 2 weeks to accelerate neutrophil recovery. Eligible patients proceeded to aHSCT. The efficacy of induction treatment was assessed by bone marrow aspiration after the recovery of blood counts according to ELN criteria [16], with complete remission (CR) defined as bone marrow blasts < 5%, absence of extramedullary disease, absolute neutrophil count ≥ 1.0 × 10^9^/L and platelet count ≥ 100 × 10^9^/L, CRi as all CR criteria except for residual neutropenia < 1.0 × 10^9^/L or thrombocytopenia < 100 × 10^9^/L, and partial remission (PR) as bone marrow blast percentage 5% to 25% or decrease in blasts in bone marrow by at least 50%. Early mortality was defined as death during or after the chemotherapy, usually within the first few weeks of treatment before discharge from the hospital. 

The statistical analysis was performed using Statistica 8.0 (StatSoft, Inc. Tulsa, OK, USA) software. The distribution of continuous variables was skewed and, thus, presented as medians. Categorical data were reported as frequencies and percentages. The differences between the compared groups were analyzed using the Mann–Whitney test. The Kaplan–Meier method was used to estimate OS. Study groups were compared by using the Gehan–Wilcoxon test. The impact of variables on OS was assessed using a multivariate Cox proportional hazard model. A *p*-value of 0.05 or less was considered statistically significant.

The study was approved by the University Medical Centre Ljubljana Ethics Committee (approval 301122/8) and followed guidelines set out in the Declaration of Helsinki.

## 3. Results

From 1 January 2012 to 31 December 2022 (11 years), 130 adult patients with r/r/secondary AML were treated according to the FLAG(-Ida) protocol. Patients’ demographic, baseline, and genetic characteristics are presented in Table 1.

Cytogenetics was performed in 129/130 patients. Mutations in *NPM1* and *FLT3* (ITD and TKD) were analyzed in 116 and 124 patients, respectively. Out of 64 patients with normal karyotypes (49%), *NPM1* and *FLT3* were analyzed in 62 and 59 patients, respectively. NGS mutation analysis was available for 12 patients, 7 of whom were without recurrent genetic aberrations defining ELN risk subgroups.

Genetic data were used for ELN risk assessment and patients were classified according to the latest recommendation [16]. In total, 51/130 (39%) patients had an adverse risk according to ELN, most of them (48/51) with unfavorable cytogenetic changes, while myelodisplasia-related gene mutations were found in the remaining three patients with normal karyotypes. In total, 26 patients had a complex karyotype, 15 of whom had also lost the *TP53* gene. In total, 12 patients had a 7q deletion or monosomy 7. In total, 4 of 9 patients with a *KMT2A* rearrangement had a translocation t(9;11), while the remaining 5 patients had unfavorable *KMT2A* rearrangements. In the remaining patients with unfavorable cytogenetics, 2 had *MECOM* rearrangements, 2 t(9;22), and 1 t(6;9). There were 23 *FLT3* (21 *FLT3-*ITD)-positive patients, 13 of whom were concurrently *NPM1*-positive. The majority of *FLT3-*ITD-positive patients had normal karyotypes (17 patients), 2 patients had complex karyotypes, and 2 patients had deletion 7q. The 20 patients at favorable risk included 3 patients with inv(16), 1 with b-ZIP *CEBPA*, and the remaining 16 were *NPM1-*positive.

In total, 41/130 (32%) patients were treated with FLAG and 89/130 (68%) with FLAG-Ida. The patients treated with FLAG-Ida were significantly younger than the patients treated with FLAG (58 vs. 62 years; *p* = 0.01). 

The indication for chemotherapy was a relapse in 48 patients (with 2 patients experiencing 2nd and 3rd relapses, respectively), resistant AML in 56 patients, and secondary AML in 26 patients. Although initially there were 32 patients with secondary AML, 6 of them exhibited resistance to prior daunorubicin–cytarabine-based chemotherapy and were consequently categorized as resistant. Patients who relapsed had a median time of relapse of 12 months after the previous occurrence of the disease. The majority of r/r patients had previously received daunorubicin–cytarabine-based chemotherapy: 75 patients DA3+7, 27 patients DA3+7+cladribine [17,18], and 2 patients received hypomethylating agents before FLAG(-Ida). 

In total, 125/130 (96%) patients had at least one episode of fever during the hospitalization, which was followed by broad-spectrum antibiotics administration. In total, 16/130 (12%) died during the hospitalization; their median age was 61.5 years, 5 had secondary AML, 4 had relapsed AML, and 7 had resistant AML. In total, 10/16 were treated with FLAG. The median time to platelet recovery above 20 × 10^9^/L and neutrophil count recovery above 0.5 × 10^9^/L was 27 days and 28 days after the first day of chemotherapy, respectively. The median time of hospitalization was 34 days. There were no significant differences in median time to platelet (27 vs. 35 days) and neutrophil count (28 vs. 29 days) recovery or hospitalization time (33 vs. 34 days) following FLAG and FLAG-Ida.

The overall response was achieved in 91/130 (70%) patients; CR in 53/130 (41%), CRi in 36/130 (28%), and PR in 2/130 (1.5%). Table 2 demonstrates the efficacy results according to different patients’ characteristics. 

After FLAG(-Ida), 50 patients continued treatment with a cytarabine-based consolidation (36 patients received another FLAG(-Ida), 10 patients high/intermediate-dose cytarabine plus mitoxantrone, and 4 patients high-dose cytarabine) and 16 patients with hypomethylating agents. In total, 61/130 (47%) patients proceeded to aHSCT. Patients who proceeded to aHSCT were significantly younger than those who did not (median age 63 vs. 52 years; *p* < 0.001).

The median overall survival (OS) time for the whole group of patients was 9.4 months. The median time of observation was 8.4 months (257 days; range 3–4051 days). 

**Figure 1:**
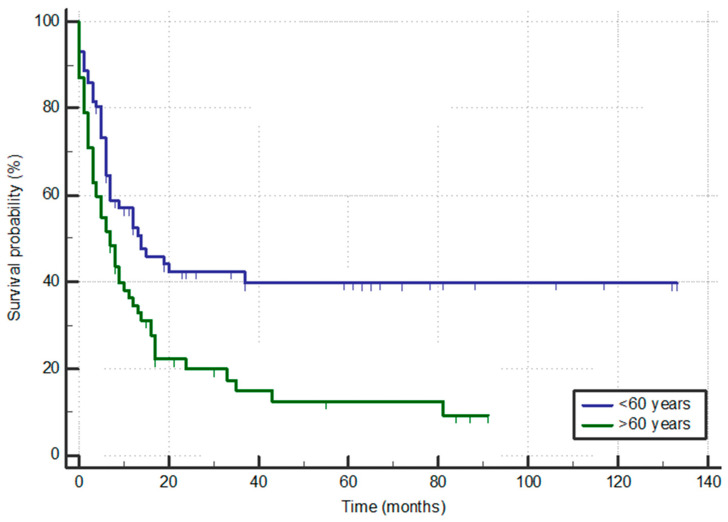
Overall survival according to age.

**Figure 2:**
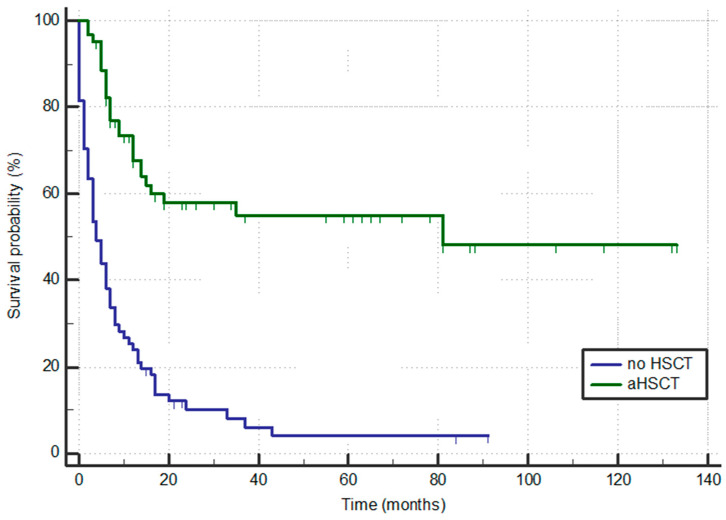
Overall survival according to allogeneic hematopoietic stem cell transplantation (aHSCT).

Patients who were older than 60 years had significantly shorter OS times compared to those up to 60 years of age (6.9 vs. 14.0 months; *p* = 0.006), as demonstrated in Figure 1. Patients who continued treatment with aHSCT had significantly prolonged OS compared to those who did not (63 months vs. 4.2 months; *p* < 0.001), as depicted in Figure 2, with 35/61 still alive at the time of analysis in contrast to only 5/69 alive among those not transplanted. 

There were no significant differences in median OS in patients treated with FLAG compared to FLAG-Ida (7.5 vs. 10.2 months; *p* = NS). 

When only patients with r/r AML (104 patients of median age 60 years) were analyzed (secondary AML excluded), the overall response rate was 74/104 (71%), and the median OS was 10.1 months. Out of the 6 patients who had secondary AML and were resistant to previous daunorubicin–cytarabine-based chemotherapy, only 2 responded to FLAG.

Patients with secondary AML (*n* = 26, median age 59 years) responded to the regimen in 65%. In total, 12/26 (46%) patients proceeded to aHSCT. The median OS of patients with secondary AML was 8.6 months.

Patients with relapsed AML (48) had the longest median OS of 16 months. The median time from the first diagnosis of AML and relapse in those patients was 12 months and there were no significant differences in OS between the 4 patients with early relapse (<6 months) or 44 patients with late relapse (6 months or more after the first diagnosis); however, the compared groups were small.

Patients with FLT3 mutation responded to FLAG/FLAG-Ida in 65%. In total, 8/23 patients proceeded to aHSCT. The median OS of FLT3-positive patients was 5.8 months compared to 12.9 months for non-FLT3-positive patients; *p* = NS. 

Patients with adverse risk according to ELN had shorter median OS times compared to the others (7.0 months vs. 12.0 months; *p* = NS). 

We performed multivariate Cox regression analysis to assess the impact of variables on OS (Table 3). Only aHSCT was significantly associated with OS.

## 4. Discussion

The results of this retrospective study focusing on r/r and secondary AML demonstrate, similarly to the findings of other researchers [10,11,19], that FLAG-Ida is a potent therapy; the overall response rate in our cohort of patients was 70%. However, a significant survival benefit was found only for patients who continued treatment with aHSCT. Furthermore, among the subgroups of r/r and secondary AML patients, the longest median OS of 16 months was observed in patients with relapsed AML, in whom the median time of relapse was 12 months. It has been shown before that patients with late relapses are more likely to respond to salvage chemotherapy and have a longer OS [3,4]. 

As expected for r/r and secondary AML, the risk categories are different to newly diagnosed AML. While the proportion of normal karyotypes is consistent with newly diagnosed AML (49%) [20], the proportion of good-risk AML is much lower, at only 14%. Slightly overrepresented is the unfavorable group (39%), which would increase if NGS analysis was performed for all patients with normal karyotypes without *NPM1* and *FLT3-*ITD mutation. Indeed, patients were not routinely screened for somatic mutations by NGS until this diagnostic procedure was routinely available. 

The high early mortality of 12% in our cohort is comparable to the data of Montesinos et al. (15%) [10] and Westhus et al. (9% at 30 days and 16% at 60 days) [11] and reflects the toxicity of the regimen causing prolonged neutropenia during which infectious complications are common. All but five of our patients had febrile episodes.

There were no statistically significant differences observed in terms of CR, OS, and the proportion of patients proceeding to aHSCT between the FLAG and FLAG-Ida regimens. This finding aligns with the study conducted by Virchis et al. [21], where the FLAG and FLAG-Ida regimens were employed for newly diagnosed high-risk AML and MDS patients. In contrast, the study conducted by Farooq et al. [22] yielded different results in their small patient cohort, indicating the superiority of FLAG over FLAG-Ida in terms of CR, survival, and transplant rate. Notably, our analysis did not reveal any significant disparities in the median time to platelet and neutrophil count recovery or in hospitalization duration following either therapy. However, patients who received FLAG-Ida were significantly younger than those who received FLAG. 

*FLT3-*ITD mutation, identified in approximately 30% of patients with newly diagnosed AML, is a strong risk factor for relapse [23,24]. *FLT3-*ITD was positive in only 21 patients (16%). The reason it was not present as frequently as expected is partially a consequence of a high rate of MDS-related cytogenetic changes in our patients (43/130, 33%) that is usually not associated with *FLT3* mutation [24]. The frequency of *FLT3-*ITD in the normal karyotype group was, however, close to expected (17/64; 27%) [24]. Gilteritinib is a *FLT3* inhibitor approved for patients with r/r *FLT3-*mutation-positive AML based on the phase 3, randomized, controlled ADMIRAL trial [25]. In this trial, the superiority of gilteritinib vs. salvage chemotherapy was reported with an overall response rate of 67.6% vs. 25.8% and OS 9.3 vs. 5.6 months, respectively. The reason for the inferior results of the salvage chemotherapy group was that only 40/109 patients received FLAG-Ida and 41/109 of the patients were treated non-intensively with azacitidine (25/109) or low-dose cytarabine (16/109). Although our *FLT3*-positive patients had better combined rates of CR and CRi (65%) compared to the salvage chemotherapy arm in the ADMIRAL trial (15%), this did not translate into a longer median OS (5.8 months). The reason was probably that the majority of these patients did not undergo aHSCT which has been demonstrated to be the most important predictor of long-term survival in the extension phase of the ADMIRAL trial [26] as well as in general in r/r AML [4]. In our opinion, a potential drawback of gilteritinib is its long and unpredictable time to respond, which can complicate aHSCT planning. Reville et al. have demonstrated that high-dose cytarabine and purine analogs mitigate the negative prognostic impact of FLT3 mutations; however, the best results might be attained when combining FLAG(-Ida) with gilteritinib followed by aHSCT [27,28].

There have been an increasing number of studies, mainly retrospective, focusing on the use of a BCL-2 inhibitor venetoclax with hypomethylating agents in the r/r setting [29]. In contrast to de novo AML, the results for r/r AML are less favorable, with overall responses ranging from 31% [30] to 64% [31] and survival rarely exceeding 6 months [29]. Similarly to patients with newly diagnosed AML, the mutational profile is important as is previous exposure to hypomethylating agents and aHSCT. *NPM1, RUNX1,* or *SRSF2* mutations have been associated with higher response rates and *FLT3-ITD, TP53,* or *DNMt3A* and complex karyotypes with worse outcomes. Responders of the therapy may also proceed to aHSCT [32,33]. The majority of studies demonstrate inferior response and survival of r/r patients undergoing low-intensity hypomethylating agent plus venetoclax compared to high-intensity regimens, also in terms of bridging to aHSCT [29]. In our patient cohort, those who were older than 60 years and, particularly, those who did not proceed to aHSCT, had significantly shorter survival. We chose the age of 60 years as a clinical division between “younger” vs. “older” patients, consistent with international and local recommendations guiding therapeutic decisions [16]. However, we demonstrate that age did not significantly affect OS; rather, younger patients were more likely to undergo aHSCT. Older patients not eligible for aHSCT would likely have benefited more from a venetoclax and hypomethylating agent combination. However, the latter treatment is not currently approved for the r/r setting. For younger and fit patients with r/r AML, current directions are focused on investigating the combinations of FLAG-Ida with 7–14 days of venetoclax demonstrating impressive results in the frontline setting, even for secondary AML [34,35]. In the r/r setting the responses were less but still promising; CR/Cri ranged from 60% to 78% [19,35,36]. DiNardo et al. reported a median OS of 13 months and the transition to aHSCT significantly improved survival [35]. 

While CPX-351 has become the standard of care for older patients with secondary AML, who might similarly benefit from a combination of azacitidine and venetoclax [2,6], for younger patients with secondary AML, the optimal treatment regimen is less clear. The median age of our patients with secondary AML was 59 years. The overall response to FLAG(-Ida) was 65%, with 46% of patients proceeding to aHSCT; this outcome is superior compared to the study by Lancet et al., who reported an overall remission rate of 48%, with 34% of patients proceeding to aHSCT after CPX-351 [6]. Our results of CR/CRi compare very well to the findings of Benitez et al. [7], who demonstrated superior response, faster neutrophil and platelet recovery, lower 30-day mortality, and similar median OS in secondary AML patients treated by purine analog plus high-dose cytarabine compared to CPX-351. Likewise, a recent UK NCRI AML19 trial [37] reported noninferiority of FLAG-Ida compared to CPX-351 in terms of response and survival in younger patients (median age 56 years) with high-risk MDS and adverse cytogenetic AML. The short OS of our patients with secondary AML (8.6 months) is within the range of findings reported in other studies [6,8,14]. On the contrary, Russell et al. [38] reported a median OS of more than 20 months following FLAG-Ida treatment in a cohort of secondary AML patients with a median age of 52 years.

HSCT in r/r AML remains the most efficient treatment providing disease control with approximately 40% long-term OS [12,39]. It was performed in 47% of our r/r/secondary AML patients and was estimated to be the only factor significantly influencing OS, among other variables, namely, *FLT3* mutation, ELN adverse risk, FLAG vs. FLAG-Ida, and age. Our transplanted patients had a median OS of more than 5 years (with 35/61 (57%) remaining alive during the observation period). Achieving CR prior to the transplant is an important prognostic factor for achieving long-term remission [40]. The treatment with FLAG(-Ida) in our patient group resulted in a relatively high response rate for second-line therapy, providing ample time to prepare eligible patients for the transplant. Favorable OS in the transplanted patients could also be attributed to our transplant approach favoring myeloablative conditioning for better disease control in all fit patients of appropriate age [41], using CLARA/BRIDGE sequential therapy in patients with resistant disease and sorafenib in patients with *FLT3-*ITD-positive AML for post-transplant maintenance therapy [42]. 

## 5. Conclusions

FLAG(-Ida) chemotherapy continues to be a viable treatment option for patients with r/r and secondary AML who are candidates for aHSCT. The combined use of FLAG-Ida and venetoclax holds promise for even more favorable outcomes compared to chemotherapy alone. However, for older and unfit patients, novel combinations of less toxic targeted drugs and small molecules offer superior options for disease control compared to intensive salvage chemotherapy. The optimal treatment approach for this patient population remains to be established.

## Figures and Tables

**Table 1:** Patients’ characteristics at the time of treatment.

Patients	*n* = 130
Age (years, range)	60 (19–79)
>60 years	61 (47%)
Male	60 (46%)
WBC at the beginning of chemotherapy (10^9^/L)	2.1 (0.19–210)
Relapsed AML	48 (37%)
Resistant AML	56 (43%)
Secondary AML	26 (20%)
Prior treatment	
aHSCT	10 (8%)
Daunorubicin–cytarabine-based chemotherapy	102 (88%)
Chemotherapy for hematological malignancy	23 (18%)
Chemotherapy for non-hematological malignancy	5 (4%)
Risk stratification according to ELN	
Adverse	51 (39%)
Intermediate	61 (47%)
Favorable	18 (14%)
Main genetic subgroups	
*FLT3*-positive AML	23 (18%); 21: *FLT3*-ITD, 4: *FLT3*-TKD; 2: both
*NPM1*-positive normal karyotype AML	30 (23%)
Complex karyotype	26 (20%)
MDS-related genetic aberrations	43 (33%)
Normal karyotype	64/130 (49%)

**Table 2:** Efficacy of FLAG(-Ida) according to patients’ characteristics.

	CR (*n*, %)	CRi (*n*, %)	PR (*n*, %)	TF (*n*, %)	Early Death ^∞^
All patients, *n* = 130	53 (41)	36 (28)	2 (1.5)	34 (26)	5 (3.8)
Relapsed AML, *n* = 48	22 (46)	15 (31)	0	10 (21)	1 (2.0)
Resistant AML, *n* = 56	25 (45)	11 (20)	1 (2.0)	17 (30)	2 (3.6)
Secondary AML, *n* = 26	6 (23)	10 (38)	1 (3.8)	7 (27)	2 (7.7)
Adverse risk (ELN2022), *n* = 51	17 (33)	15 (29)	1 (2.0)	16 (31)	2 (3.9)
Intermediate risk (ELN2022), *n* = 61	26 (43)	17 (28)	1 (1.6)	14 (23)	3 (4.9)
Favorable risk (ELN2022), *n* = 18	10 (56)	4 (22)	0	4 (22)	0
FLT3-positive AML, *n* = 23	10 (43)	4 (17)	1 (4.3)	6 (26)	2 (8.7)
Treated with FLAG-Ida, *n* = 89	38 (43)	28 (31)	2 (2.2)	18 (20)	3 (3.4)
Treated with FLAG, *n* = 41	15 (37)	8 (20)	0	16 (39)	2 (4.9)

^∞^ died before assessment of response, CR = complete remission, CRi = complete remission with incomplete hematologic recovery, PR = partial response, TF = treatment failure.

**Table 3:** Variables affecting overall survival in our patients.

	Hazard Ratio	*p*	95% CI
Age	1.00	0.58	0.99–1.00
FLAG vs. FLAG-Ida	0.84	0.45	0.53–1.33
Adverse risk according to ELN	1.53	0.06	0.98–2.37
*FLT3* mutation	1.65	0.08	0.95–2.85
aHSCT	0.24	<0.001	0.14–0.43

## Data Availability

The data presented in this study are available on request from the corresponding author. The data are not publicly available due to ethical restrictions.
